# Changes in metabolism affect expression of ABC transporters through ERK5 and depending on p53 status

**DOI:** 10.18632/oncotarget.23305

**Published:** 2017-12-14

**Authors:** Sana Belkahla, Abrar Ul Haq Khan, Delphine Gitenay, Catherine Alexia, Claire Gondeau, Dang-Nghiem Vo, Stefania Orecchioni, Giovanna Talarico, Francesco Bertolini, Guillaume Cartron, Javier Hernandez, Martine Daujat-Chavanieu, Nerea Allende-Vega, Martin Villalba Gonzalez

**Affiliations:** ^1^ Department of Lymphocyte Differentiation, Tolerance and Metabolism: Basis for Immunotherapy, Institut De Médecine Régénératrice Et Biothérapie (IRMB), INSERM, Univ De Montpellier, Montpellier, France; ^2^ Department of Lymphocyte Differentiation, Tolerance and Metabolism: Basis for Immunotherapy, Institut De Médecine Régénératrice Et Biothérapie (IRMB), CHU Montpellier, Montpellier, France; ^3^ Département d'Hépato-gastroentérologie A, Hôpital Saint Eloi, CHU Montpellier, Montpellier, France; ^4^ Department of Oncology and Hemato-Oncology, European Institute of Oncology, Milan, Italy; ^5^ Département d'Hématologie Clinique, CHU Montpellier, Université Montpellier I, Montpellier, France; ^*^ These two authors share senior authorship

**Keywords:** ABC transporter, p53, ERK5, oxidative phosphorylation (OXPHOS), dichloroacetate (DCA)

## Abstract

Changes in metabolism require the efflux and influx of a diverse variety of metabolites. The ABC superfamily of transporters regulates the exchange of hundreds of substrates through the impermeable cell membrane. We show here that a metabolic switch to oxidative phosphorylation (OXPHOS), either by treating cells with dichloroacetate (DCA) or by changing the available substrates, reduced expression of ABCB1, ABCC1, ABCC5 and ABCG2 in wild-type p53-expressing cells. This metabolic change reduced histone changes associated to active promoters. Notably, DCA also inhibited expression of these genes in two animal models *in vivo*. In contrast, OXPHOS increased the expression of the same transporters in mutated (mut) or null p53-expressing cells. ABC transporters control the export of drugs from cancer cells and render tumors resistant to chemotherapy, playing an important role in multiple drug resistance (MDR). Wtp53 cells forced to perform OXPHOS showed impaired drug clearance. In contrast mutp53 cells increased drug clearance when performing OXPHOS. *ABC* transporter promoters contain binding sites for the transcription factors MEF2, NRF1 and NRF2 that are targets of the MAPK ERK5. OXPHOS induced expression of the MAPK ERK5. Decreasing ERK5 levels in wtp53 cells increased ABC expression whereas it inhibited expression in mutp53 cells. Our results showed that the ERK5/MEF2 pathway controlled ABC expression depending on p53 status.

## INTRODUCTION

The main purpose of cell metabolism is the conversion of metabolic substrates to energy (catabolism) or to build blocks for generating new molecules (anabolism). Metabolism requires the intake of substrates and the outtake of metabolites through the highly impermeable cell plasma membrane and multiple transporters with differences in specificity have been described. One of the biggest and better-conserved families of transporters is the ATP-binding cassette (ABC transporters [1, 2]). The ABC superfamily has 48 members that have been classified into 7 groups (subfamilies A to G). One of their main biological functions is the transport of lipids [3], essential molecules for cell metabolism. Although it is conceivable that changes in metabolism regulate ABC expression, this remains uninvestigated.

ABC transporters are well known for their role in multiple drug resistance (MDR) because many of them export anticancer drugs and render tumors resistant to chemotherapy [4]. The tumor suppressor gene *p53* is an important regulator of metabolic homeostasis by promoting OXPHOS and inhibiting glycolysis [5]. It induces the expression of different metabolic genes, such as cytochrome c oxidase 2 (*SCO2*), glutaminase 2 (*GLS2*), p53 up-regulated modulator of apoptosis (*PUMA*), glucose transporter 1 and 4 (*GLUT1* and *GLUT4*) and TP53-induced glycolysis and apoptosis regulator (*TIGAR*) [6, 7]. More than half of all human tumors harbor mutations in the *p53* gene. Most of these mutations abrogate its DNA binding and transactivation activity [8], but others can provide mutant p53 (mutp53) with gain-of-function (GOF) activity that is dependent on its *de novo* ability to regulate directly or indirectly gene expression [9, 10]. Furthermore, certain p53 mutants enhance drug resistance in liver cancer cells and B-CLL cells [11, 12].

Several *ABC* promoters contain consensus p53 binding sequences [4]. The direct regulation of ABC promoters by p53 is controversial and depends on the architecture of the promoter, the nature of p53 (mutant or wild type), the presence of other p53 family members and variations cell- and tissue-specific [4, 13]. We have observed that inducing OXPHOS in leukemic cells sensitizes wtp53-expressing cells to genotoxic drugs such as doxorubicin and vincristine [7]. In contrast, cells carrying mutp53 were more resistant to these anticancer drugs.

OXPHOS promotes the expression of the MAPK extracellular signal-regulated kinase-5 (ERK5), which regulates the choice of metabolic substrates in hematopoietic cells [14–19]. ERK5 induces activation of several members of the MEF2 family of transcription factors by different mechanisms. It mediates direct serine and threonine phosphorylation of MEF2A, C and D [20, 21]. It activates MEF2A and D by direct interaction because ERK5 serves as a MEF2 coactivator through its signal-dependent direct association with the MEF2 MADS domain [22, 23]. The effect of activated ERK5 on MEF2A-dependent transcription requires ERK5 kinase activity. MEF2 mediates several ERK5 effects on metabolism, including NRF2 activation [15, 16]. This transcription factor and the related NRF1 are master regulators of metabolism that are coordinated with MEF2 family to induce expression of metabolic genes [24]. The promoters, or very proximal regions, of several *ABC* genes, i.e. ABCB1, ABCB4, ABCB8, ABCB9, ABCB10, ABCB11, ABCC1, ABCC4, ABCC12 and ABCG1, have MEF2A binding sites that have been validated by ChIP (http://genome.ucsc.edu/). Most of these transporters also have NRF1 or NRF2 binding sites, i.e. ABCB8, ABCB9, ABCB10, ABCB11, ABCC1, ABCC3, ABCC5, ABCC4, ABCC10, ABCC12, ABCC13, ABCG1, which have also been validated by ChIP (http://genome.ucsc.edu/). This information is based on ChIP-seq experiments performed by the ENCODE consortium[25]. Hence, we investigated here if metabolism, through ERK5, controls ABC expression and the role of p53 status.

## RESULTS

### OXPHOS regulated ABC transporters in AML cell lines

**Figure 1:**
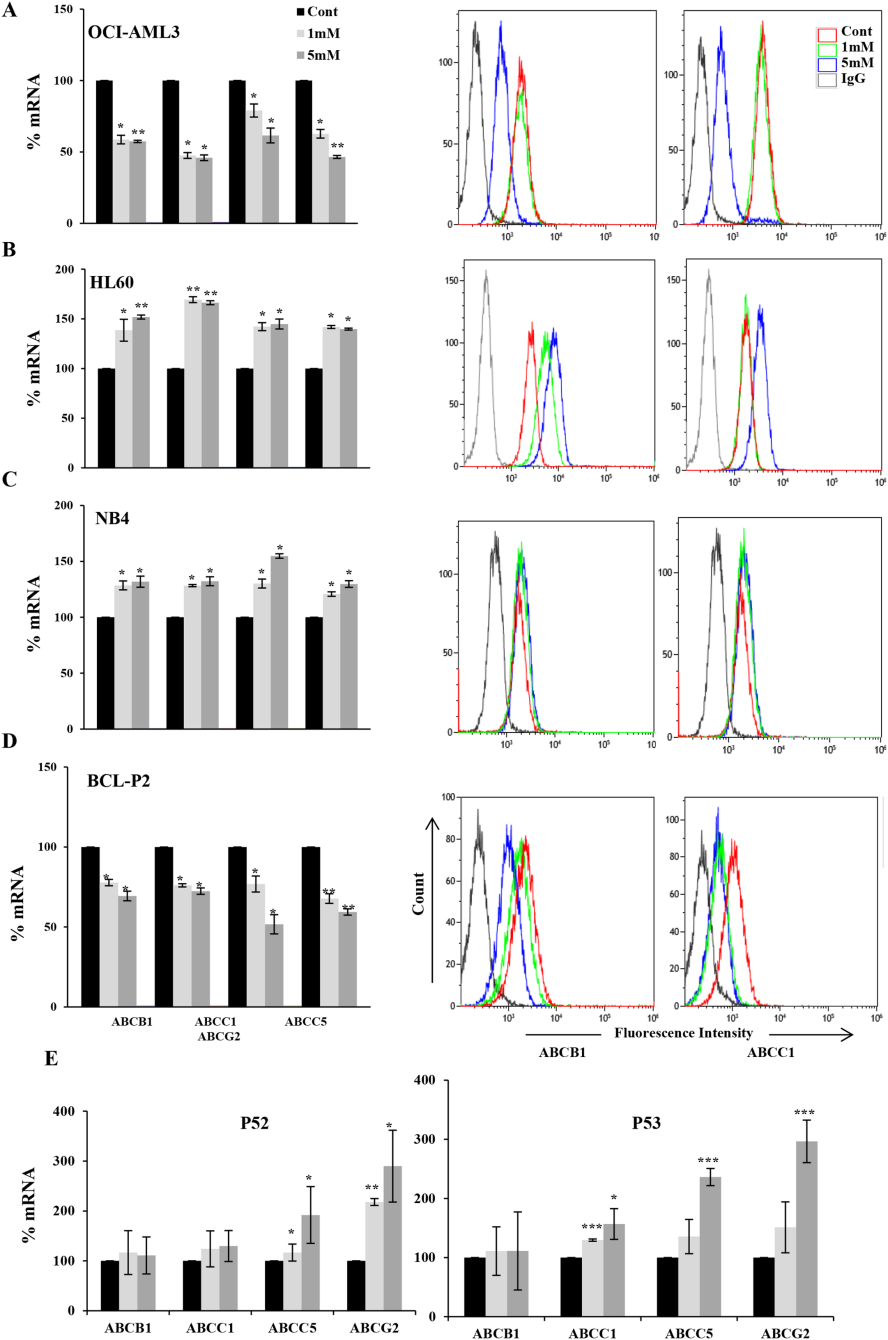
DCA-induced ABC transporters expression depended on p53 status in leukemic cells Different hematopoietic cell lines OCI-AML3 wtp53 **(A)**, HL-60 nullp53 **(B)**, NB4 mutp53 **(C)** and primary cells from BCL-P2 wtp53 patient **(D)** or B-CLL mutp53 patients were treated with 1 and 5 mM DCA for 1 week and RNA and plasma protein levels were analyzed by qPCR or FACS respectively. Data represent the % of mRNA compared to control cells. The bar graphs represent means ± SD of 3 independent experiments performed in triplicate; ^*^p<0.05, ^**^p<0.01, ^***^p<0.005 student t-test compare to control cells.

We induced OXPHOS either by inhibiting pyruvate dehydrogenase kinase (PDK) with dichloroacetate (DCA; [7, 14, 16, 26–28]) or by incubating cells in the absence of glucose. When glucose is no longer available, cells use alternative energy substrates, such as glutamine (Gln). Gln oxidation, or glutaminolysis, generates ATP through OXPHOS [29, 30] and this pathway is functional in leukemic cells [15, 31]. We called OXPHOS medium a glucose-free medium supplemented with 10 mM galactose and 4 mM Gln [14, 15, 31]. OXPHOS induces expression of both wild type (wt) and mutp53 depending on the cellular context [7]. We used DCA concentrations on the range of that found in plasma of DCA-treated patients [32, 33] and tested the effect on the expression of 4 ABC transporters, ABCB1, ABCC1, ABCC5 and ABCG2, involved in MDR, in 3 AML cell lines with different p53 status: OCI-AML3 cells express wt p53, HL60 are p53 null and NB4 are mutp53 [7]. DCA decreased by half the mRNA of the different ABC transporters on OCI-AML3 cells and this correlated with protein reduction in the 2 examined transporters (Figure 1A). In contrast, in HL-60 and NB4 cells, DCA increased mRNA and protein expression (Figure 1B and 1C).

Consequently with our observations in cell lines, we observed that DCA decreased *ABC* mRNA and protein expression in primary cells derived from a B-cell lymphoma (BCL) patient with wtp53 (Figure 1D). On the contrary DCA increased expression of *ABCC5* and *ABCG2* in two mutp53 patients and *ABCC1* in one patient. In contrast, *ABCB1* expression was unchanged (Figure 1E). Due to lack of enough patient samples, we could not analyze protein expression.

**Figure 2:**
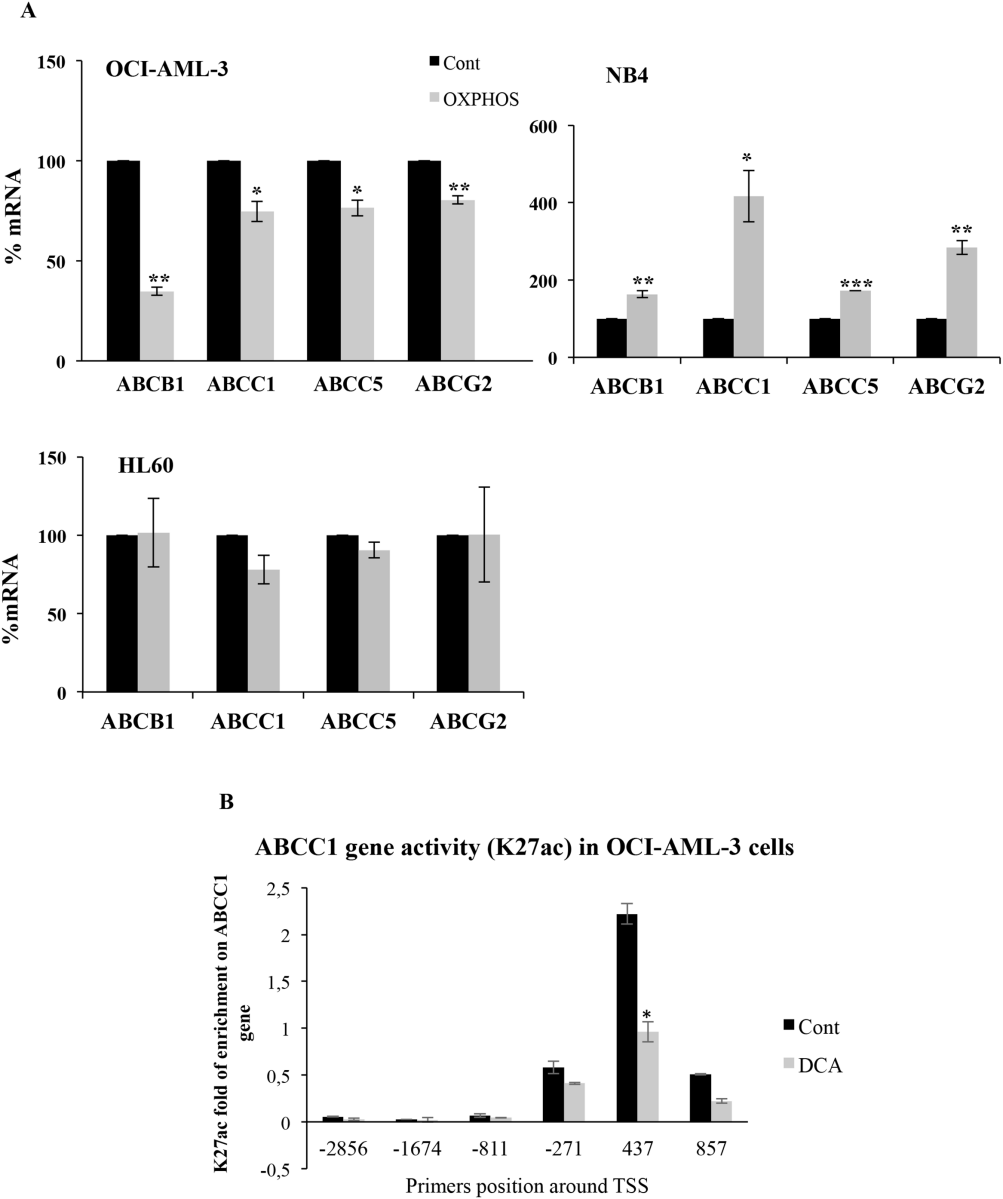
OXPHOS inhibited or stimulated ABC expression in wtp53 or mutp53 cells, respectively **(A)** OCI-AML3, NB4 and HL60 cells were grown in OXPHOS medium for 2 weeks and expression of ABC transporters was analyzed as in Figure 1. The bar graphs represent means ± SD of 3 independent experiments performed in triplicate; ^*^p<0.05, ^**^p<0.01, ^***^p<0.005 student t-test compare to control cells. **(B)** Cells were treated with 5 mM DCA for 3 days and processed for ChIP analysis. The enrichment of K27 acetylation on Histone 3 in the *ABCC1* promoter was analyzed as described in material and methods by using different primers around the transcription start site (TSS).

Moreover, OCI-AML3 cells growing in OXPHOS medium displayed reduced mRNA expression of the above-mentioned ABC transporters (Figure 2A). Conversely, mutant p53-expressing NB4 cells increased *ABC* expression in the same medium (Figure 2A). In contrast to DCA, OXPHOS medium did not increase ABC expression in null p53 HL60 cells (Figure 2A). This showed that although DCA and OXPHOS medium could have similar effects, they could also induce different outcomes depending on p53 status. This could be related to the different stimulation periods needed to optimize changes in cell metabolism.

To verify that OXPHOS was affecting transcription of these genes, we assessed histone modifications that have been linked to *ABCC1* promoter activation [11, 12] in OCI-AML3 cells. We found that DCA inhibited acetylation on H3 K27 on the *ABCC1* promoter (Figure 2B). We conclude that DCA inhibited transcription of *ABC* transporters on wtp53 cells.

### OXPHOS regulated ABC transporters in hepatic cells

**Figure 3:**
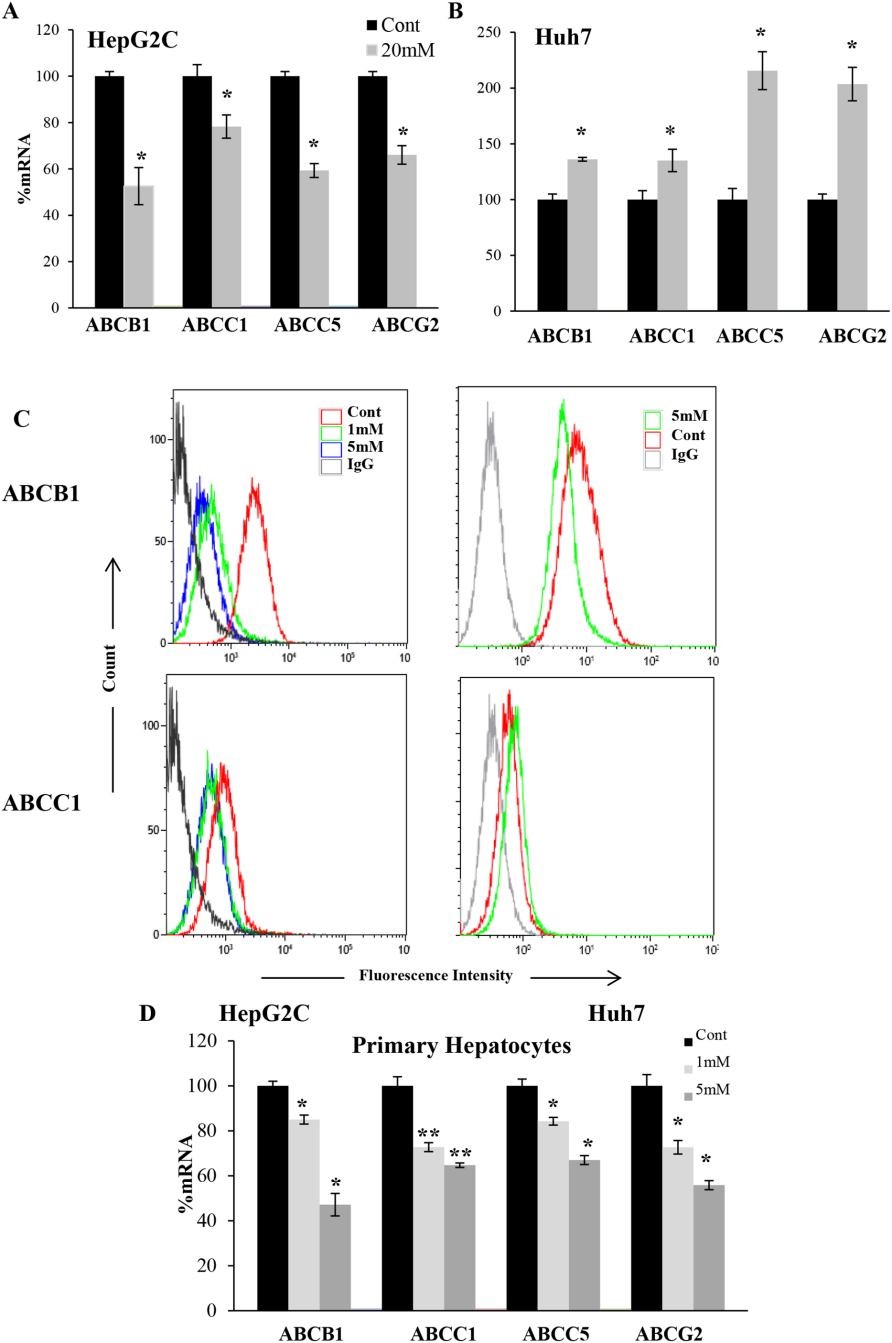
DCA-induced ABC transporters expression depended on p53 status in hepatic cells **(A-B)** Hepatic cell lines HepG2-C3A wtp53 and HuH7 mutp53 were treated with 20mM DCA for 24h before analyzing mRNA level as described in Figure 1. **(C)** Cells were treated with the indicated concentration of DCA for 1 week and protein level was analyzed by FACS. **(D)** Primary hepatocytes were treated with indicated amount of DCA for 72 hours. The data represent means ± SD (n=3); ^*^p < 0.05, ^**^p < 0.01, ^***^p < 0.001 Student's t-test compared to control cells or as depicted in the graphic.

The main detoxifying organ is liver. We tested DCA effect in 2 hepatic cell lines expressing wtp53 (HepG2-C3A) or mutp53 (HuH7). When wtp53 was present, an acute dose of DCA decreased *ABC* transporters mRNA and protein (Figure 3A and 3C). In contrary, and similar to mutp53 leukemia cells, DCA increased *ABC* mRNA expression in HuH7 cells and also ABCC1 protein (Figure 3B and 3C). Surprisingly, ABCB1 protein decreased after treatment suggesting alternative posttranscriptional regulation. Finally, we examined the effect of OXPHOS on ABC transporters expression in non-transformed cells. Primary hepatocytes from non-cancer patients, which were obviously wtp53, showed the same pattern than cell lines expressing wtp53 (Figure 3D).

### DCA decreased mRNA of *ABC* transporters *in vivo*

**Figure 4:**
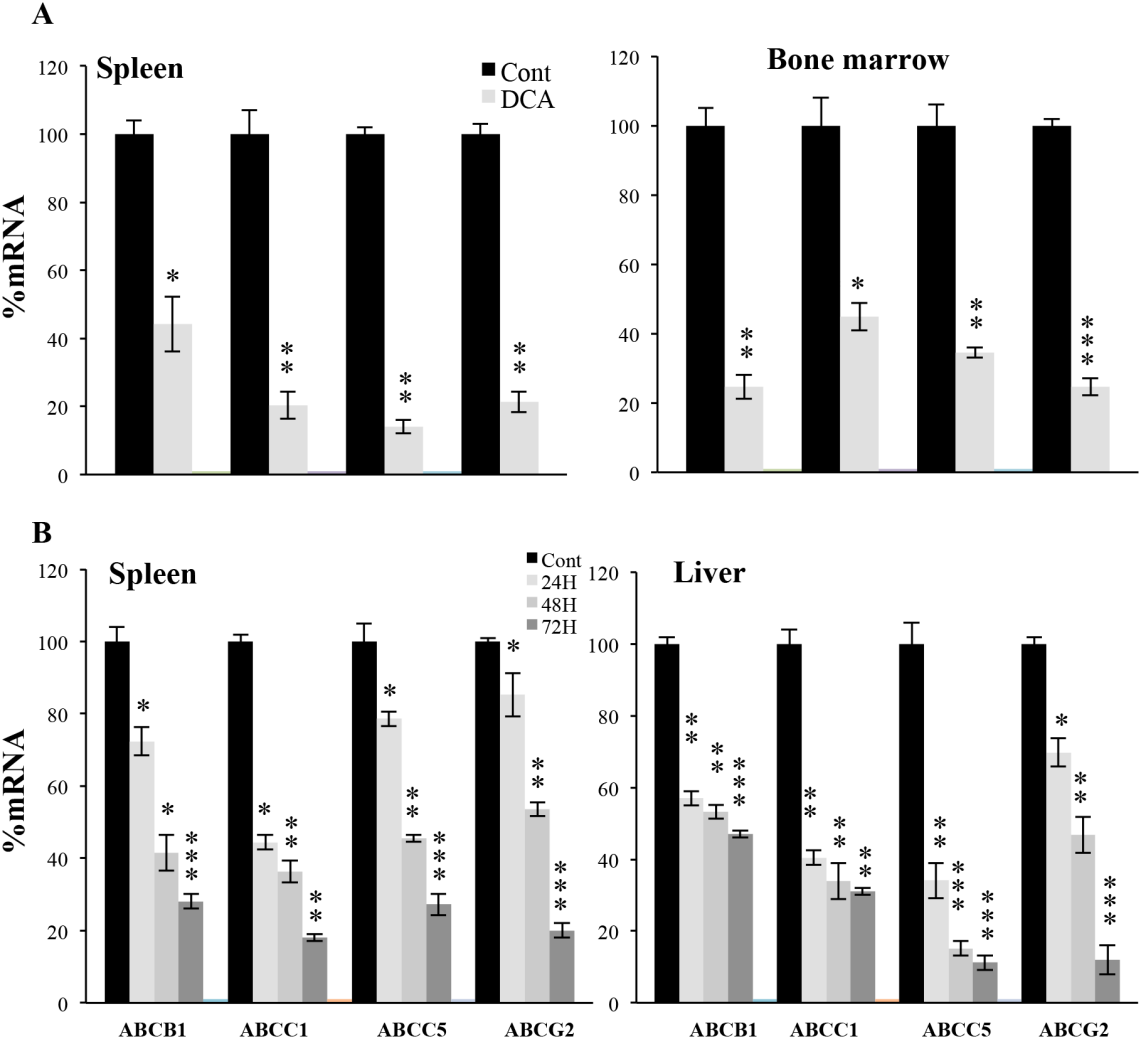
DCA induced ABC transcription *in vivo* **(A)** NSG mice were engrafted with primary human AML cells. At day 80 post-graft, they were treated with DCA (n=4) or leave untreated (n=4). At day 140, mRNA from spleen or bone marrow was isolated and human ABCB1, ABCC1, ABCC5 and ABCG2 mRNA expression was quantified by qPCR. **(B)** B6 wt mice (n=4/5 per group) were treated with a dose of DCA (50 mg/kg) everyday intraperitoneally and mice mRNA in spleen and liver at different times was obtained to analyze *ABC* transporters. The data represent means ± SD; ^*^p<0.05, ^**^p<0.01, ^***^p<0.001 student t-test compare to non treated cells or mice.

We had observed that DCA increased doxorubicin efficacy *in vivo* on tumor cells expressing wtp53 [7]. We engrafted human wtp53 AML primary cells in non-obese diabetic/severe combined immunodeficient (NOD/SCID)-interleukin-2 receptor γ chain null (NSG) mice, as previously described [7, 16]. Mice with established tumors (day 80 post-graft) were treated daily with DCA. The treatment was not toxic and did not show any notable effect on mice survival [7]. Human tumor AML cells gather in mouse spleen and bone marrow, hence we isolated mRNA from these organs and used human-specific primers. We observed that DCA significantly reduced expression of *ABC* transporters mRNA (Figure 4A).

Additionally, we assessed the effect of DCA in normal tissues of non-tumor bearing wt mice. A daily injection of DCA, for 1 and up to 3 days, also reduced mouse *ABC* transporters mRNA in liver and spleen (Figure 4B). The effect largely increased with the number of doses received. Hence, DCA reduced *ABC* expression in multiple wtp53 cell populations *in vivo*. This could, at least partially, explain its effect on genotoxic drug treatment in wtp53 tumor cells *in vivo* [7].

### OXPHOS modulated drug clearance from tumor cells depending on its p53 status

**Figure 5:**
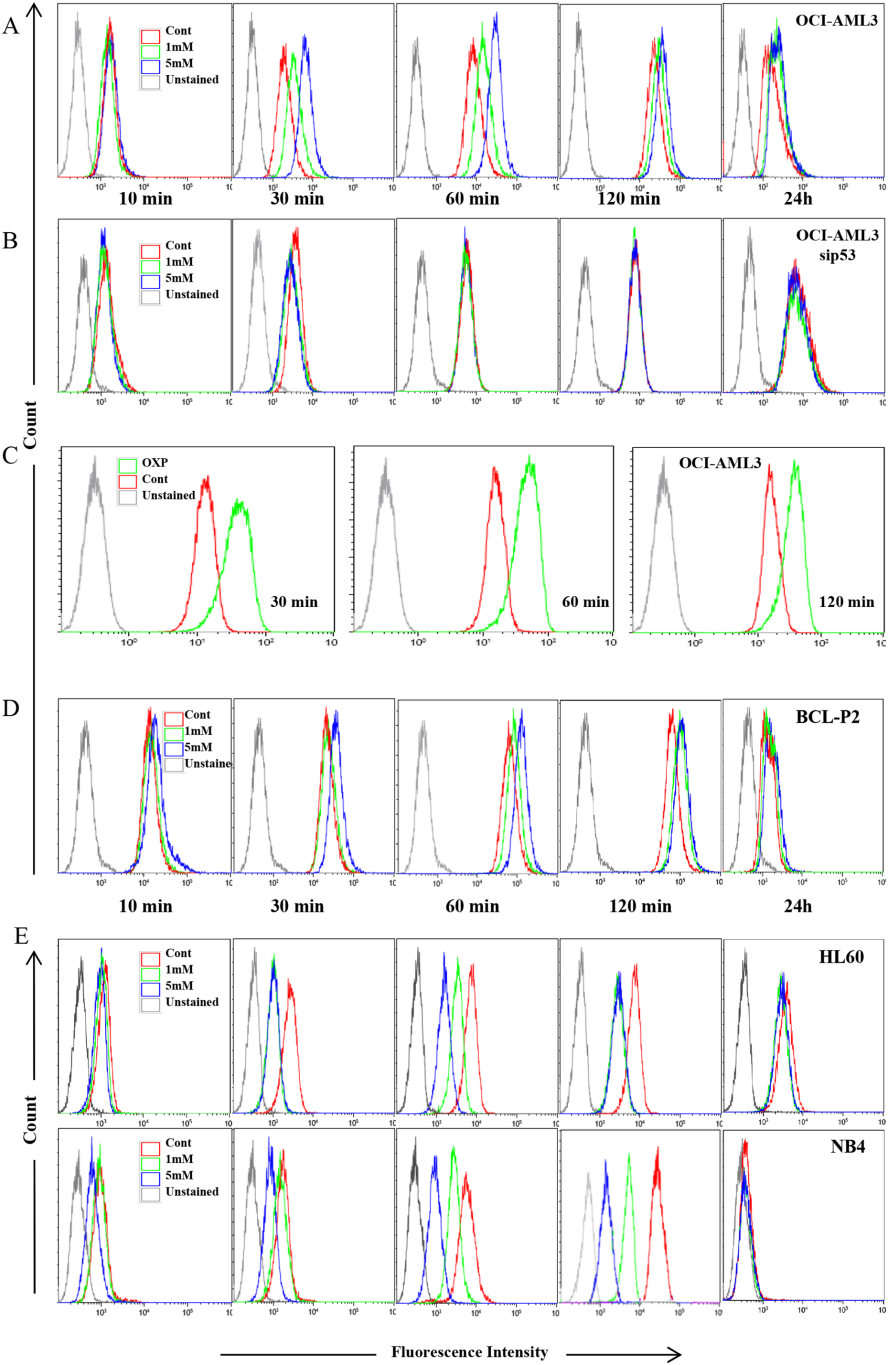
Metabolism-controlled drug outtake depended on p53 status **(A, B, D** and **E)** Different cell lines were treated with 5 mM DCA for 7 days or incubated in OXPHOS medium for 14 days (C). In (B), OCI-AML3 cells were transfected with a siRNA for p53 (sip53) 48 h before treatment. Cells were then incubated with 5 uM daunorubicin and clearance was measured at various time points (10, 30, 60, 120 minutes). Some cells were incubated with 1uM daunorubicin for 24 h. Experiments were repeated at least three times.

A direct functional consequence of modulating ABC transporters expression in tumor cells could be changes in their responsiveness to chemotherapeutic drugs. To investigate if the changes in the expression of ABC transporters affect drug clearance, we incubated tumor cells with the genotoxic drug daunorubicin, which is a fluorescent compound. This allows monitoring its intracellular accumulation by FACs. In addition, it is used in the clinic to treat leukemia and lymphoma, which are at the origin of some cell lines that we have used in our study. Finally, like other anthracyclines, it is exported from cells by a broad range of transporters [34]. We used OCI-AML3 cells treated for one week with 1 mM and 5mM DCA or growing in OXPHOS medium. Then, cells were further incubated with daunorubicin and drug clearance was followed by FACs. We found that OXPHOS OCI-AML3 cells were impaired on decreasing drug levels (Figure 5A and 5C). The effect was mainly observed at short time points, but it was still noticeable as much as 24 h after (Figure 5A). Primary wtp53 tumor cells from a BCL patient also showed decreased daunorubicin export (BCL-P2; Figure 5D). As expected, the outcome was opposite in cells with alterations in p53. In tumor cells not expressing p53 (HL-60) or expressing mutp53 (NB4) DCA induced a faster drug clearance (Figure 5E) in agreement with an increase on ABC transporters. Furthermore, blocking expression of wtp53 in OCI-AML3 cells by treating them with a small interference RNA against p53 (sip53), which effectively decreased p53 expression (Supplementary Figure 1), prevented DCA-induced decreased daunorubicin clearing (Figure 5B), whereas a control siRNA did not show any effect (data not shown). Therefore, changes in mRNA and/or protein expression of ABC transporters due to metabolic changes lead to alterations in drug clearance, which depended in p53 status.

**Figure 6:**
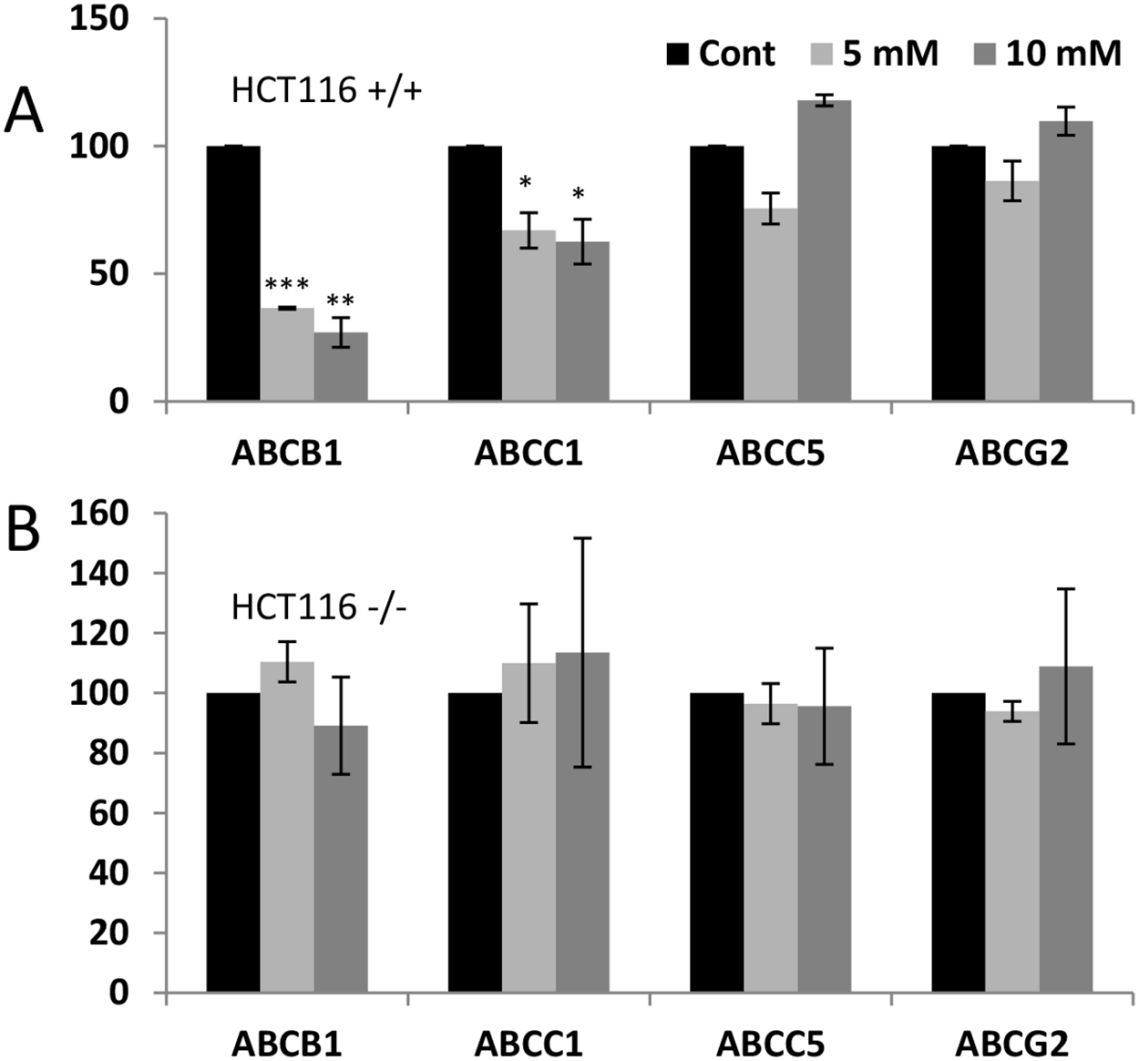
DCA-induced ABC transporters expression required wtp53 **(A** and **B)** Two isogenic colon cancer cell lines (p53^+/+^ and p53^-/-^ HCT116 cells) were treated with 1 and 5 mM DCA for 1 week and RNA was analyzed by qPCR. Data represent the % of mRNA compared to control cells. The bar graphs represent means ± SD of 3 independent experiments performed in triplicate; ^*^p<0.05, ^**^p<0.01, ^***^p<0.005 student t-test compare to control cells.

To further investigate the role of p53 in ABC transporter expression after metabolic changes, we used two isogenic colon cancer cell lines (p53^+/+^ and p53^-/-^ HCT116 cells) that differently respond to DCA treatment [7]. DCA decreased the *ABCB1* and *ABCC1* mRNAs without changing those of *ABCC5* and *ABCG2* (Figure 6A). p53^-/-^ HCT116 cells did not downregulate any *ABC* mRNA after DCA treatment (Figure 6B). These results from HCT116^+/+^ cells suggest that the role of p53 in ABC expression is cell type dependent and depends in multiple factors [4, 13]. The absence of DCA effect on HCT116^-/-^ and of OXPHOS medium in null p53 HL60 cells, suggest that p53 is essential to mediate the effect of metabolism on ABC expression.

### ERK5 was essential for DCA-induced effects

**Figure 7:**
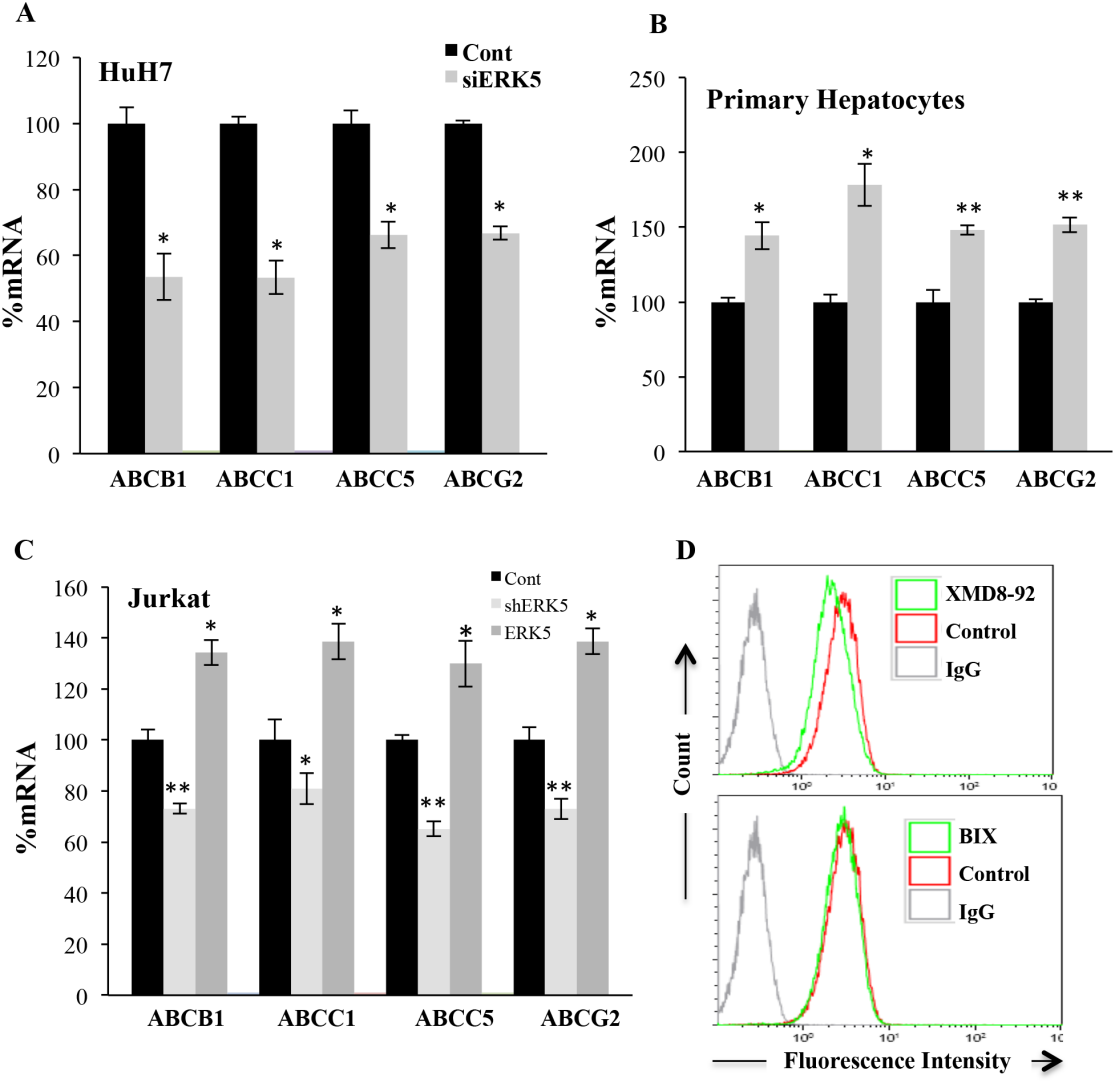
The ERK5 pathway regulated ABC transcription **(A)** HuH7 cells were transfected with control siRNA or siERK5. Thirty-six hours later mRNA expression was analyzed by qPCR. **(B)** Primary human hepatocytes were transfected with control siRNA or siERK5 at day 1 and 3 post seeding. 96 h later mRNA levels were assessed. **(C)** Jurkat cells were transfected with a small hairpin RNA for ERK5 (shERK5) or ERK5 expression vector. 36h later mRNA expression was analyzed by qPCR. **(D)** Jurkat cells were treated with 10 μM XMD8-92 or 10 μM BIX01289 for 24 h before analyzing expression of ABCB1 by FACs. The data represent means ± SD n=3; ^*^p < 0.05, ^**^p < 0.01, ^***^p < 0.001 Student's t-test compared to empty vector transfected cells (control).

We further investigated the underlying mechanism of OXPHOS-induced ABC transporters expression. We focused on the role of the ERK5/MEF2 pathway, which is activated in cells performing OXPHOS [14–16, 31]. However, we could not reduce ERK5 levels in cells performing OXPHOS because this kinase is essential for cell survival in this metabolic status [14–16, 31]. We speculated that decreasing ERK5 levels in non-treated cells would have the opposite effect than DCA treatment. Reducing expression of ERK5 with a small interference RNA for ERK5 (siERK5), which reduced ERK5 levels (Supplementary Figure 2A and 2B), decreased and increased *ABC* transporters mRNA in HuH7 and primary hepatocytes, respectively (Figure 7A and 7B). This suggested that ERK5 effects, like those of DCA, depended on p53 status. To test this hypothesis, we used Jurkat cells that express very low levels of mutp53 [35]. DCA or OXPHOS medium induced a small increase in ABCB1 protein (Supplementary Figure 3). Consequently a shERK5, which encodes for a different sequence that the previous described siRNA and decreased ERK5 expression (Supplementary Figure 2C), decreased mRNA of multiple *ABC* transporters (Figure 7C). In agreement, the ERK5 inhibitor XMD8-92 or the MEK5 inhibitor BIX02189 also decreased basal ABCB1 protein expression (Figure 7D). Finally, overexpression of ERK5 (Supplementary Figure 2C) increased *ABC* mRNA expression (Figure 7C).

## DISCUSSION

ABC transporters have attracted great attention in recent years due to their role in MDR [4]. But, it should be kept in mind that their main physiological role in eukaryotes is possibly the transport of other substances out of the cell, e.g. lipid transport [3]. It is somehow intuitive that changes in metabolism regulate the transport of a variety of molecules. We show here that wtp53 cells, which comprise the vast majority of non-transformed cells, while performing OXPHOS decrease expression of ABC transporters. During OXPHOS, cells use most of the carbon of metabolic substrates to produce energy and CO2. Then, they should avoid releasing metabolites to external media. In contrast, during glycolysis, cells generate a number of intermediate metabolites that can not accumulate and would need to be exported. We hypothesize that this is the physiological interest to increase expression of the exporter ABC transporters during high glycolysis. However, tumor cells take advantage of this fact to acquire MDR. Transformed cells present a metabolism more orientated to anaerobic glycolysis than their non-transformed counterparts, which mainly use respiration/OXPHOS. This should favor tumor MDR by increasing ABC transporters activity in wtp53 expressing cells. In fact, MDR1-dependent chemoresistant cells show an even more glycolytic metabolism that the sensitive counterparts [36, 37]. Interestingly, hypoxia or changes in glucose levels increase MDR1 expression by hypoxia-inducible factor-1 (HIF-1; [38, 39]). This could partly explain the observation that hypoxic tumor cells are more chemoresistant [13]. These observations could be exploited in the clinic to prevent MDR. Since more glycolytic tumors show higher MDR, inducing a metabolic switch should decrease MDR, although as we prove here the p53 status should be taken into account.

High expression of ABC transporters is often observed in drug-naive tumors compared with the tissue of origin and, as previously described, this correlates with a more glycolytic metabolism. Hence, constitutive MDR1 or MRP over expression is likely regulated in some cells by pathways involved in malignant transformation, e.g. metabolic changes [13]. Metabolic drugs such as DCA could revert tumor metabolism and decrease ABC transporters expression. However, DCA accumulates both wt and mutp53 protein [7]. If the last induces ABC expression as suggested in several cell lines by our results and in previous works [4, 13], DCA would finally increase MDR. HSP90 inhibitors, e.g. 17-AAG, degrade mutp53 by favoring its MDM2-mediated ubiquitination [40], and we have shown that DCA cooperates with 17-AAG to kill mutp53 tumor cells [7]. This could be a possibility to treat mutp53 tumors. In summary, it is essential to know p53 status before treatment with metabolic drugs as we have previously shown [7].

ERK5 could use different ways to transcriptionally activate *ABC* promoters, e.g. p53, NF-κB, NRF2, HIF1 and MEF2 [13]. ERK5 inhibition induces p53 activation and increases sensitivity to 5-fluorouracil in colon cancer cells but the role of ABC was not investigated [41]. We propose that ERK5 activity by regulating metabolism affects p53 effect in *ABC* promoters.

Oxidative stress induces expression of ABC transporters, which is likely mediated by NRF2 [42, 43]. ERK5, through MEF2, generates an antioxidant response independently of de novo ROS generation through NRF2 activation [16]. Hence, by activating NRF2, ERK5 could modulate ABC expression.

HIF regulates ABC expression [38, 39] and ERK5 regulates in normoxia an array of genes regulated by HIF1 in hypoxia [44]. In fact, ERK5 by modulating metabolism regulates genes that are regulated by HIF. This could be the case of *ABC* genes. Indeed, NF-κB mediates ABC expression by direct binding to *ABC* promoters [13, 45] or by regulating intermediate mediators. In particular during changes in glucose levels, NF-κB activates HIF1 that targets ABC promoters [39]. Given the fact that ERK5 activates NF-κB [46], this could mediate the effect of metabolism on ABC expression.

As previously described, the promoters of several *ABC* genes studied here, i.e. ABCB1 and ABCC1, have MEF2A binding sites. MEF2 mediates a large part of the observed ERK5 effects on metabolism [15, 16, 47]. Hence, this could also be the case of ABC transporters. The existence of multiple ERK5 targets mediating ABC expression makes it difficult to elucidate their individual relevance. The contribution of each factor could depend, as discussed for p53 [4, 13], on the architecture of the promoter, the level of expression of each target, the presence of other targets and variations cell- and tissue-specific.

ERK5 inhibition in mutp53 cells decreased ABC expression (our results) and should decrease MDR and favors chemotherapy as it has been shown [41], although the mechanism was not described. Our results show that metabolism targets multiple *ABC* promoters, depending on p53 status, which will have a clear effect on drug export. In this context, DCA overcomes resistance to paclitaxel in A549/taxol cells [48]; however, p53 status is unknown in this subline although the parental cell line, i.e. A549, is wtp53. Then DCA could decrease expression of ABC transporters in this study as we have observed here. However, Zhou et al proposed that ABCB1 expression did not change and expression of other ABC members was not analyzed [48]. Authors propose that citric acid accumulation induces apoptosis, but DCA and paclitaxel can also activate alternative pathways leading to cell death. In our case, we have shown that inducing OXPHOS in wtp53 cells cooperates with genotoxic drugs to eliminate tumor cells *in vitro* and *in vivo* [7].

An interesting clinical approach would be to treat patients whose tumors express wtp53 with DCA before standard chemotherapy. We have used here DCA concentrations similar to that used in patients [7, 32, 33]. These concentrations were effective to change ABC expression and then should sensitize tumor cells to chemotherapy as we have observed in mice [7]. In fact a combinatorial therapy involving DCA in preclinical settings have been proposed with several compounds such as metformin [49], Nutlin-3 [50] or paclitaxel [51]. But until our knowledge there is a current lack of clinical results.

The control of ABC transporters by cell metabolism is a physiologic process required to optimize substrate use. Therefore wtp53 cells in proliferative states, which enter aerobic glycolysis, would also increase ABC expression and become more resistant to drug treatment. This could have implications for the treatment of non-cancerous proliferative conditions. For example, in coronary artery stent restenosis and pulmonary artery hypertension (PAH) there is an unwanted proliferation of endothelial cells. Notably, inhibition of ABCC4 prevents and reverses pulmonary hypertension in mice [52] and ABCA3 deficiency is related to PAH of the newborn [53]. Moreover, ABCG2 clears hypoxia-induced intracellular metabolites and protects the heart from pressure overload-induced ventricular dysfunction [54]. Hypoxia-induced aerobic glycolysis could obviously affect the effectiveness of drug treatment in these situations. This could be also the case in other situations requesting high proliferation such as inflammation or wound healing.

In summary, the effects of metabolism on the transcription of ABC transporters clearly depend on p53 status. However, sensitization to chemotherapy by metabolic drugs does not depend only on ABC transcription because pro-apoptotic pathways are also activated that could lead to synergism between metabolic drugs and standard chemotherapy.

## MATERIALS AND METHODS

### Ethical statement

Experimental procedures were conducted according to the European guidelines for animal welfare (2010/63/EU). Protocols were approved by the Animal Care and Use Committee “Languedoc-Roussillon” (approval number: CEEA-LR-12163).

### Reagents and antibodies

DCA was from Santa Cruz Technologies. Galactose and glutamine were from GIBCO. Human anti-ABCB1, ABCC1 and control IgG1 were from Miltenyi Biotec and 7AAD from Beckman. The ERK5 inhibitor XMD8-92 and the MEK5 inhibitor BIX02189 were from Selleck.

### Plasmids

The expression vectors for ERK5, the pSUPER expression vector for GFP alone or GFP plus shERK5 have been previously described [46]. Control, ERK5 and p53 siRNA were ON-TARGETplus SMARTpools (mixture of 4 siRNA) from Dharmacon and has been previously used [7, 47]. ERK5 shRNA (AGCTGCCCTGCTCAAGTCT) was transfected in Jurkat cells as previously described [46].

### *In vivo* mouse experiments

*In vivo* experiments were carried out using 6 to 8 weeks/old male NSG mice. Mice were bred and housed in pathogen-free conditions in the animal facility of the European Institute of Oncology–Italian Foundation for Cancer Research (FIRC), Institute of Molecular Oncology (Milan, Italy). For engraftment of human cells, 1 million AML cells were injected intravenously (i.v.) through the lateral tail vein in non-irradiated mice. NSG mice with established human AML tumors (day 80 post-graft) were treated with DCA (50 mg/kg, 1 dose/day by gavage, starting at day 1 for 16 consecutive days). Human tumor AML cells gather in mouse spleen and bone marrow, hence we isolated mRNA from these organs. We used human-specific primers to visualize expression of human *ABC* mRNA. In a different experiment B6 wt mice were treated in pathogen-free conditions at the INM animal facility at Montpellier, France, with a daily single dose of DCA (50 mg/kg/day) intraperitoneally and mouse LDLR mRNA was analyzed in spleen and liver after different times.

### Cell lines and culture conditions

The leukemic human cell lines, HL-60, NB4, Jurkat TAg and OCI-AML3 were grown in RPMI 1640–Glutamax (GIBCO) supplemented with 5% (Jurkat) or 10% (OCI) FBS [15, 16]. Primary cells from a lymphoma B cell patient (BCL-P2) were grown in the same medium with 10% FBS. In certain experiments cells were grown in RPMI 1640 without glucose (GIBCO 11879) with the addition of 2 mM glutamine and 10 mM galactose (OXPHOS medium). The Jurkat TAg cells carry the SV40 large T Ag to facilitate cell transfection. The hepatic cell lines HepG2-C3A and HuH7 were grown in MEM and DMEM respectively supplemented with 10% FBS, 1 mM sodium pyruvate, 2 mM glutamine, penicillin and streptomycin. The HCT116 human colon cancer cells were cultured in low glucose (5 mM) DMEM medium supplemented with 10% FBS. Cellular confluence during experiments was between 80-85% for adherent cells.

### Human liver samples and preparation of PHHs cultures

Liver samples were obtained from liver resections performed in adult patients for medical reasons (CRB-CHUM - Biological Resource Center of the Montpellier University Hospital, Dr. Jeanne Ramos and Pr. Sylvain Lehmann). The use of human specimens for scientific purposes was approved by the French National Ethics Committee. All methods were carried out in accordance with the approved guidelines and regulations of this committee. Written informed consent was obtained from each patient prior to surgery. Human hepatocytes isolation and culture were performed as described previously [55]. Briefly, after liver perfusion, hepatocytes were counted and cell viability was assessed by trypan blue exclusion test. A suspension of 1x10^6^ cells/mL per well was added in 12-well plates pre-coated with type I collagen (Beckton Dickinson) and cells were allowed to attach for 12h. Then, the supernatant containing dead cells and debris was carefully removed and replaced with 1 mL of serum-free long-term culture medium (Lanford medium, LNF). The number of confluent attached cells was estimated at ∼1.5x10^5^ cells/cm^2^.

### Transient transfection

Jurkat cells in logarithmic growth phase were transfected with the indicated amounts of plasmid by electroporation [46, 56]. In each experiment, cells were transfected with the same total amount of DNA by supplementing with empty vector. Cells were incubated for 10 min at RT with the DNA mix and electroporated using the Gene Pulser Xcell™ Electroporation system (Bio-Rad) at 260 mV, 960 mF in 400 μl of RPMI 1640. Expression of the different proteins was confirmed by western blot. The transfection efficiency in Jurkat TAg cells is between 60 and 80%. In HuH7 cells, transfection of 30–50 nM siRNAs was carried out using Lipofectamine RNAiMAX (Invitrogen) in Opti-MEM (Invitrogen), according to the manufacturer’s instructions. Primary hepatocytes were transfected twice, at day first and third post-seeding. Cells were harvested 48 to 96 h post-transfection. OCI-AML3 cells (2 x 10^6^ in 100 μl) were transfected with 300nM p53 siRNA, or control siRNA by electroporation using Nucleofactor Electroporation system (Lonza). Cells were harvested 24 to 72 h post-transfection.

### Counting and determination of cell viability

Cell viability and cell numbers were determined using the Muse^®^ Cell Analyzer (Millipore) Number of cells means number of live cells and viability is the number of live cells counted divided by total cell counted (alive and dead cells).

### RT-PCR

Total RNA was extracted using NucleoSpin RNA isolation columns (Macherey-Nagel). Reverse transcription was carried out using iScript™ cDNA Synthesis Kit (Biorad). Quantitative PCR was performed with KAPA SYBR Green qPCR SuperMix (Cliniscience) and a CFX Connect™ Real-Time qPCR machine (Biorad) with ABCB1, ABCC1, ABCC5, ABCG2 and actin primers. All samples were normalized to β-actin mRNA levels. Results are expressed relative to control values arbitrarily set at 100. The primers used were:

### Mouse primers

ABCB1: Forward: (5′-TTCTCTTTGTCCGCGGAGTC-3′) Reverse: (5′- GAATGCTTCCAGGCATAAGCG-3′), ABCC5: Forward: (5′- CTAGCTGGTCGTTTCACGGT -3′) Reverse: (5′-CCTCCTGCAGCCCACTATAC-3′), ABCC1: Forward: (5′-CAAAGCCGGTGGAAAATGGG-3′) Reverse: (5′- GTGGGAAGACGAGTTGCTGA-3′), ABCG2: Forward: (5′- CTCCTTGCCAGATAAGAGGGG-3′) Reverse: (5′- CCTCAGTTAATTTCAGGACGACAG-3′), Actin: Forward: (5′-GCGGACTGTTAGTGAGCTGCG-3′) Reverse: (5′- TGTTTGCTCCAACCAACTGCTGTC-3′).

### Human primers

ABCB1: Forward: (5′-GGAGGCCAACATACATGCCT-3′) Reverse: (5′- AGGCTGTCTAACAAGGGCAC-3′), ABCC5: Forward: (5′- CTTGTTTTGCTGCAGGGCTC-3′) Reverse: (5′-CACATCAGAATTCCTGCGCC -3′), ABCC1: Forward: (5′-CCCGCTCTGGGACTGGAA-3′) Reverse: (5′- GTAGAAGGGGAAACAGGCCC -3′), ABCG2: Forward: (5′- TGTGTTTATGATGGTCTGTTGGTC ′) Reverse: (5′- TGTTGCATTGAGTCCTGGGC -3′), Actin: Forward: (5′-GAGGGAAATCGTGCGTGACA-3′) Reverse: (5′-AATAGTGATGACCTGGCCGT-3′).

### Flow cytometry

Briefly, 1x10^6^ cells were stained with antibody in PBS with 2% FBS and incubated at 37°C for 30 min. Cells were then washed and suspended in 200–250 μl PBS 2% FBS and staining was analyzed using a Gallios flow cytometer (Beckman) and the Kaluza software.

### ChIP experiment

OCI-AML3 cells were treated with 10mM DCA for 72 h. Ten million cells were centrifuged (5min; 1200rpm) and the pellet was washed two times in 1X phosphate-buffered saline (PBS) at room temperature and suspended in 10mL of 1X PBS. Cells were fixed in 1% paraformaldehyde (Electron Microscopy Sciences) at room temperature for 5 min and lysed in 1 ml of cell lysis buffer (5 mM PIPES, 85 mM KCl, 0.5% NP40, Na Butyrate 10mM + 2X protease inhibitor cocktail (Halt™ Protease Inhibitor Cocktail, EDTA-Free (100X), Thermofischer)) at 0°C for 10 min. Nuclei were recovered by centrifugation (10min, 5000rpm) at 4°C and lysed in 250μl nuclei lysis buffer (50 mM Tris-HCl pH 7.5, 1% SDS, 10 mM EDTA, Na Butyrate 10mM + Halt™ Protease Inhibitor Cocktail (3X)) at 4°C for at least 2 hours. 250μl of each sample were then sonicated 2 times for 5 min (30 s on/off) at 4°C using a Bioruptor (Diagenode). After sonication, absorbances at 280 nm (A280) of 1/100 diluted samples were measured and A280nm and was adjusted to 0.133 with nuclei lysis buffer. One hundred microliter were used for ChIP experiments in a final volume of 1 ml. Samples were incubated under gentle agitation at 4°C overnight in the presence of 3μg of either a specific antibody or a negative control. Antibodies (anti-K27Ac Ab4729 (Abcam) and negative control IgG (Diagenode)) were previously bound to DYNA Beads Protein G Novex (Life Technology) according to the supplier’s recommendations. Dynabeads-bound immunoprecipitates were sequentially washed once with a low salt buffer (50 mM Tris-HCl pH 7.5, 150 mM NaCl, 1% triton, 0.1% SDS, 1 mM EDTA, 1mM Na Butyrate + Halt™ Protease Inhibitor Cocktail (1X)), a high-salt buffer (50 mM Tris-HCl pH 7.5, 500 mM NaCl, 1% triton, 0.1% SDS, 1 mM EDTA, 1mM Na Butyrate) and a LiCl-containing buffer (20 mM Tris-HCl pH 7.5, 250 mM LiCl, 1% NP40, 1% Na deoxycholate, 1 mM EDTA, 1mM Na Butyrate) and, then, twice with a TE buffer (10 mM Tris-HCl pH 7.5, 1 mM EDTA, Tween 20 0.02%). Samples were then eluted in 250μL of elution buffer (100 mM NaHCO3, 1% SDS), and DNA-protein complexes were incubated at 65°C for 5 hours to reverse crosslinks. Samples were then treated with 100mg/ml proteinase K and 100 mg/ml RNAse A at 45°C for 2 hours to digest proteins and contaminating RNA. DNA was purified with an extraction kit (NucleoSpin Gel and PCR clean-up, Macherey-Nagel) according to the manufacturer’s recommendations and qPCR analysis was performed using the Roche LightCycler 480 real-time PCR system. The data were normalized with inputs taken from samples before the immunoprecipitation and treated under the same conditions. The primers used to amplify various regions of LDLR gene promoter. Supplementary Table 1 shows the primers used in this experiment.

### Daunorubicin accumulation / drug uptake Studies

The intracellular daunorubicin accumulation in cells was examined by flow cytometer [57]. The logarithmically growing cells were cultured in 48-well plates and incubated with or without 5 μM daunorubicin for different times (10', 30', 60', 120' minutes) and 1 μM for 24 h). After incubation, cells were placed on ice, washed twice with PBS and analyzed by flow cytometry (Beckman-coulter, Elite), excitation 488 nm (argon laser) for the mean fluorescence intensity (MFI) of intracellular daunorubicin. A minimum of 10000 events was analyzed for each histogram.

### Statistical analysis

The statistical analysis of the difference between means of paired samples was performed using the paired t test. The results are given as the confidence interval (^*^: p<0.05, ^**^: p<0.01, ^***^: p<0.005). All the experiments described in the figures with a quantitative analysis have been performed at least three times in duplicate. Other experiments were performed three times with similar results.

## SUPPLEMENTARY MATERIALS FIGURES AND TABLE



## References

[1] Jones PM, George AM (2004). The ABC transporter structure and mechanism: perspectives on recent research. Cell Mol Life Sci.

[2] Ponte-Sucre A (2007). Availability and applications of ATP-binding cassette (ABC) transporter blockers. Appl Microbiol Biotechnol.

[3] Neumann J, Rose-Sperling D, Hellmich UA (2017). Diverse relations between ABC transporters and lipids: an overview. Biochim Biophys Acta.

[4] Bush JA, Li G (2002). Cancer chemoresistance: the relationship between p53 and multidrug transporters. Int J Cancer.

[5] Gottlieb E, Vousden KH (2010). p53 regulation of metabolic pathways. Cold Spring Harb Perspect Biol.

[6] Puzio-Kuter AM (2011). The role of p53 in metabolic regulation. Genes Cancer.

[7] Allende-Vega N, Krzywinska E, Orecchioni S, Lopez-Royuela N, Reggiani F, Talarico G, Rossi JF, Rossignol R, Hicheri Y, Cartron G, Bertolini F, Villalba M (2015). The presence of wild type p53 in hematological cancers improves the efficacy of combinational therapy targeting metabolism. Oncotarget.

[8] Hollstein M, Sidransky D, Vogelstein B, Harris CC (1991). p53 mutations in human cancers. Science.

[9] Muller PA, Vousden KH (2013). p53 mutations in cancer. Nat Cell Biol.

[10] Muller PA, Vousden KH (2014). Mutant p53 in cancer: new functions and therapeutic opportunities. Cancer Cell.

[11] Chan KT, Lung ML (2004). Mutant p53 expression enhances drug resistance in a hepatocellular carcinoma cell line. Cancer Chemother Pharmacol.

[12] Sturm I, Bosanquet AG, Hermann S, Guner D, Dorken B, Daniel PT (2003). Mutation of p53 and consecutive selective drug resistance in B-CLL occurs as a consequence of prior DNA-damaging chemotherapy. Cell Death Differ.

[13] Scotto KW (2003). Transcriptional regulation of ABC drug transporters. Oncogene.

[14] Charni S, de Bettignies G, Rathore MG, Aguilo JI, van den Elsen PJ, Haouzi D, Hipskind RA, Enriquez JA, Sanchez-Beato M, Pardo J, Anel A, Villalba M (2010). Oxidative phosphorylation induces de novo expression of the MHC class I in tumor cells through the ERK5 pathway. J Immunol.

[15] Lopez-Royuela N, Rathore MG, Allende-Vega N, Annicotte JS, Fajas L, Ramachandran B, Gulick T, Villalba M (2014). Extracellular-signal-regulated kinase 5 modulates the antioxidant response by transcriptionally controlling Sirtuin 1 expression in leukemic cells. Int J Biochem Cell Biol.

[16] Khan AU, Rathore MG, Allende-Vega N, Vo DN, Belkhala S, Orecchioni S, Talarico G, Bertolini F, Cartron G, Lecellier CH, Villalba M (2016). Human leukemic cells performing oxidative phosphorylation (OXPHOS) generate an antioxidant response independently of reactive oxygen species (ROS) production. EBioMedicine.

[17] Villalba M, Rathore MG, Lopez-Royuela N, Krzywinska E, Garaude J, Allende-Vega N (2013). From tumor cell metabolism to tumor immune escape. Int J Biochem Cell Biol.

[18] Villalba M, Lopez-Royuela N, Krzywinska E, Rathore MG, Hipskind RA, Haouas H, Allende-Vega N (2014). Chemical metabolic inhibitors for the treatment of blood-borne cancers. Anticancer Agents Med Chem.

[19] Catalán E, Charni S, Aguiló JI, Enríquez JA, Naval J, Pardo J, Anel A, Villalba M (2015). MHC-I modulation due to metabolic changes regulates tumor sensitivity to CTL and NK cells. Oncoimmunology.

[20] Kato Y, Kravchenko VV, Tapping RI, Han J, Ulevitch RJ, Lee JD (1997). BMK1/ERK5 regulates serum-induced early gene expression through transcription factor MEF2C. EMBO J.

[21] Kato Y, Zhao M, Morikawa A, Sugiyama T, Chakravortty D, Koide N, Yoshida T, Tapping RI, Yang Y, Yokochi T, Lee JD (2000). Big mitogen-activated kinase regulates multiple members of the MEF2 protein family. J Biol Chem.

[22] Kasler HG, Victoria J, Duramad O, Winoto A (2000). ERK5 is a novel type of mitogen-activated protein kinase containing a transcriptional activation domain. Mol Cell Biol.

[23] Yang CC, Ornatsky OI, McDermott JC, Cruz TF, Prody CA (1998). Interaction of myocyte enhancer factor 2 (MEF2) with a mitogen-activated protein kinase, ERK5/BMK1. Nucleic Acids Res.

[24] Ramachandran B, Yu G, Gulick T (2008). Nuclear respiratory factor 1 controls myocyte enhancer factor 2A transcription to provide a mechanism for coordinate expression of respiratory chain subunits. J Biol Chem.

[25] Consortium EP (2012). An integrated encyclopedia of DNA elements in the human genome. Nature.

[26] Stacpoole PW, Greene YJ (1992). Dichloroacetate. Diabetes Care.

[27] Stacpoole PW (1989). The pharmacology of dichloroacetate. Metabolism: clinical and experimental.

[28] Michelakis ED, Webster L, Mackey JR (2008). Dichloroacetate (DCA) as a potential metabolic-targeting therapy for cancer. Br J Cancer.

[29] Reitzer LJ, Wice BM, Kennell D (1979). Evidence that glutamine, not sugar, is the major energy source for cultured HeLa cells. J Biol Chem.

[30] Rossignol R, Gilkerson R, Aggeler R, Yamagata K, Remington SJ, Capaldi RA (2004). Energy substrate modulates mitochondrial structure and oxidative capacity in cancer cells. Cancer Res.

[31] Rathore MG, Saumet A, Rossi JF, de Bettignies C, Tempe D, Lecellier CH, Villalba M (2012). The NF-kappaB member p65 controls glutamine metabolism through miR-23a. Int J Biochem Cell Biol.

[32] Stacpoole PW, Kurtz TL, Han Z, Langaee T (2008). Role of dichloroacetate in the treatment of genetic mitochondrial diseases. Adv Drug Deliv Rev.

[33] Michelakis ED, Sutendra G, Dromparis P, Webster L, Haromy A, Niven E, Maguire C, Gammer TL, Mackey JR, Fulton D, Abdulkarim B, McMurtry MS, Petruk KC (2010). Metabolic modulation of glioblastoma with dichloroacetate. Sci Transl Med.

[34] Zhou Y, Wang R, Chen B, Sun D, Hu Y, Xu P (2016). Daunorubicin and gambogic acid coloaded cysteamine-CdTe quantum dots minimizing the multidrug resistance of lymphoma *in vitro* and *in vivo*. Int J Nanomed.

[35] Cheng J, Haas M (1990). Frequent mutations in the p53 tumor suppressor gene in human leukemia T-cell lines. Mol Cell Biol.

[36] Broxterman HJ, Pinedo HM, Kuiper CM, Schuurhuis GJ, Lankelma J (1989). Glycolysis in P-glycoprotein-overexpressing human tumor cell lines. Effects of resistance-modifying agents. FEBS Lett.

[37] Staubert C, Bhuiyan H, Lindahl A, Broom OJ, Zhu Y, Islam S, Linnarsson S, Lehtio J, Nordstrom A (2015). Rewired metabolism in drug-resistant leukemia cells: a metabolic switch hallmarked by reduced dependence on exogenous glutamine. J Biol Chem.

[38] Chen J, Ding Z, Peng Y, Pan F, Li J, Zou L, Zhang Y, Liang H (2014). HIF-1alpha inhibition reverses multidrug resistance in colon cancer cells via downregulation of MDR1/P-glycoprotein. PLoS One.

[39] Seebacher NA, Richardson DR, Jansson PJ (2015). Glucose modulation induces reactive oxygen species and increases P-glycoprotein-mediated multidrug resistance to chemotherapeutics. Br J Pharmacol.

[40] Li D, Marchenko ND, Schulz R, Fischer V, Velasco-Hernandez T, Talos F, Moll UM (2011). Functional inactivation of endogenous MDM2 and CHIP by HSP90 causes aberrant stabilization of mutant p53 in human cancer cells. Mol Cancer Res.

[41] Pereira DM, Simoes AE, Gomes SE, Castro RE, Carvalho T, Rodrigues CM, Borralho PM (2016). MEK5/ERK5 signaling inhibition increases colon cancer cell sensitivity to 5-fluorouracil through a p53-dependent mechanism. Oncotarget.

[42] Ji L, Li H, Gao P, Shang G, Zhang DD, Zhang N, Jiang T (2013). Nrf2 pathway regulates multidrug-resistance-associated protein 1 in small cell lung cancer. PLoS One.

[43] Wang X, Campos CR, Peart JC, Smith LK, Boni JL, Cannon RE, Miller DS (2014). Nrf2 upregulates ATP binding cassette transporter expression and activity at the blood-brain and blood-spinal cord barriers. J Neurosci.

[44] Schweppe RE, Cheung TH, Ahn NG (2006). Global gene expression analysis of ERK5 and ERK1/2 signaling reveals a role for HIF-1 in ERK5-mediated responses. J Biol Chem.

[45] Breier A, Gibalova L, Seres M, Barancik M, Sulova Z (2013). New insight into p-glycoprotein as a drug target. Anticancer Agents Med Chem.

[46] Garaude J, Cherni S, Kaminski S, Delepine E, Chable-Bessia C, Benkirane M, Borges J, Pandiella A, Iniguez MA, Fresno M, Hipskind RA, Villalba M (2006). ERK5 activates NF-kappaB in leukemic T cells and is essential for their growth *in vivo*. J Immunol.

[47] Khan AU, Allende-Vega N, Gitenay D, Gerbal-Chaloin S, Gondeau C, Vo DN, Belkahla S, Orecchioni S, Talarico G, Bertolini F, Bozic M, Valdivielso JM, Bejjani F (2017). The PDK1 inhibitor dichloroacetate controls cholesterol homeostasis through the ERK5/MEF2 pathway. Sci Rep.

[48] Zhou X, Chen R, Yu Z, Li R, Li J, Zhao X, Song S, Liu J, Huang G (2015). Dichloroacetate restores drug sensitivity in paclitaxel-resistant cells by inducing citric acid accumulation. Mol Cancer.

[49] Voltan R, Rimondi E, Melloni E, Gilli P, Bertolasi V, Casciano F, Rigolin GM, Zauli G, Secchiero P (2016). Metformin combined with sodium dichloroacetate promotes B leukemic cell death by suppressing anti-apoptotic protein Mcl-1. Oncotarget.

[50] Agnoletto C, Melloni E, Casciano F, Rigolin GM, Rimondi E, Celeghini C, Brunelli L, Cuneo A, Secchiero P, Zauli G (2014). Sodium dichloroacetate exhibits anti-leukemic activity in B-chronic lymphocytic leukemia (B-CLL) and synergizes with the p53 activator Nutlin-3. Oncotarget.

[51] Wang M, Liao C, Hu Y, Qinwen P, Jiang J (2017). Sensitization of breast cancer cells to paclitaxel by dichloroacetate through inhibiting autophagy. Biochem Biophys Res Commun.

[52] Belleville-Rolland T, Sassi Y, Decouture B, Dreano E, Hulot JS, Gaussem P, Bachelot-Loza C (2016). MRP4 (ABCC4) as a potential pharmacologic target for cardiovascular disease. Pharmacol Res.

[53] Kunig AM, Parker TA, Nogee LM, Abman SH, Kinsella JP (2007). ABCA3 deficiency presenting as persistent pulmonary hypertension of the newborn. J Pediatr.

[54] Nagy BM, Nagaraj C, Egemnazarov B, Kwapiszewska G, Stauber RE, Avian A, Olschewski H, Olschewski A (2017). Lack of ABCG2 leads to biventricular dysfunction and remodeling in response to hypoxia. Front Physiol.

[55] Pichard L, Raulet E, Fabre G, Ferrini JB, Ourlin JC, Maurel P (2006). Human hepatocyte culture. Methods Mol Biol.

[56] Garaude J, Farras R, Bossis G, Charni S, Piechaczyk M, Hipskind RA, Villalba M (2008). SUMOylation regulates the transcriptional activity of JunB in T lymphocytes. J Immunol.

[57] Venne A, Li S, Mandeville R, Kabanov A, Alakhov V (1996). Hypersensitizing effect of pluronic L61 on cytotoxic activity, transport, and subcellular distribution of doxorubicin in multiple drug-resistant cells. Cancer Res.

