# Supplementary material for: Changes in metabolism affect expression of ABC transporters through ERK5 and depending on p53 status
Source: Oncotarget. 2017 Dec 14;9(1):1114–29. doi: 10.18632/oncotarget.23305 (PMC5787424; doi:10.18632/oncotarget.23305)
Supplement: Supplementary file 1 [file oncotarget-09-1114-s001.pdf]

Changes in metabolism affect expression of ABC transporters through ERK5 and depending on p53 status

SUPPLEMENTARY MATERIALS

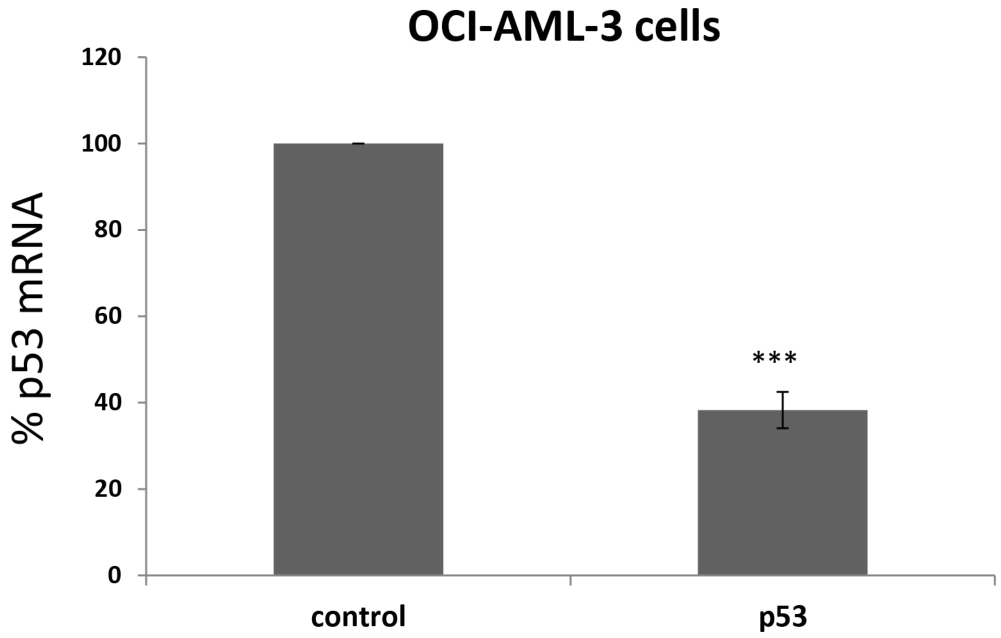

Supplementary Figure 1: OCI-AML3 cells were transfected with a siRNA for p53 and 72 h later p53 expression was analyzed by qPCR.

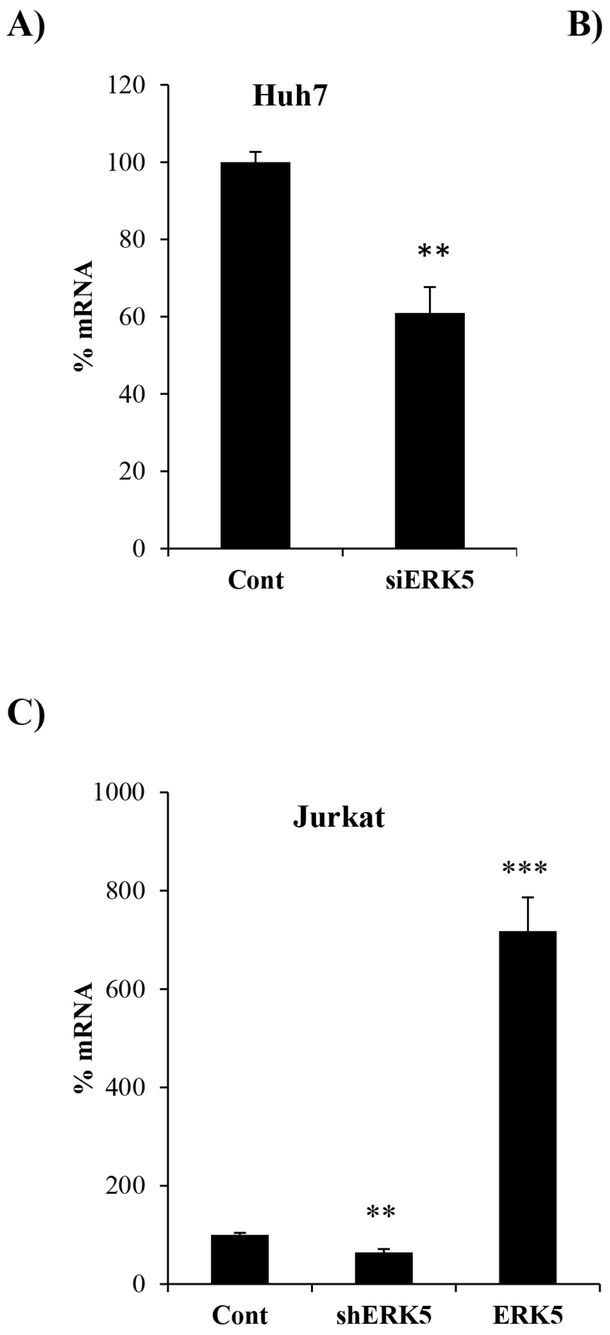

Supplementary Figure 2: (A) Huh7 cells were transfected with siERK5. **(B)** Primary Hepatocytes transfected with siERK5. **(C)** Jurkat Cells transfected with shERK5 or ERK5. 72 h later ERK5 mRNA was analyzed by qPCR.

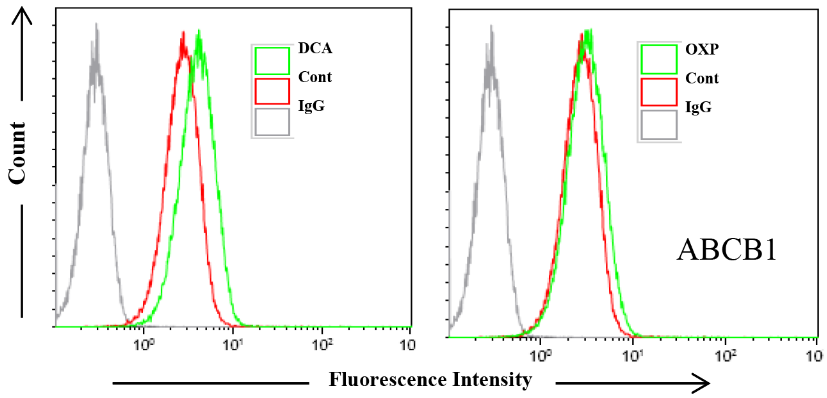

Supplementary Figure 3: OXPHOS induced ABCB1 expression on Jurkat cells. Cells were treated with 5 mM DCA for 7 days or grown in OXPHOS medium for 2 week and protein levels were analyzed by FAC.

Supplementary Table 1: Primers used for the ChIP analysis of the ABCC1 promoter on Figure 2B

OLIGONAME	SEQUENCE
MRP1-(-)2956-F	taaaacccaccagtggttcc
MRP1-(-)2956-R	gagcaggtggggtcagaag
MRP1-(-)1674-F	cttagaaactcattcacccttgg
MRP1-(-)1674-R	aaaggacctagcgagggaag
MRP1-(-)811-F	catgcctggcctacgattat
MRP1-(-)811-R	aggttattgatcctcacctcaga
MRP1-(-)271-F	tttcccctggtgacggatac
MRP1-(-)271-R	gaagcgcctgggatctttgg
MRP1-(+)437-F	agttcctgcggagcagag
MRP1-(+)437-R	gacccgaccctccaaaac
MRP1-(+)857-F	ttgatgtgccctacctgacc
MRP1-(+)857-R	agggcaggaccacagctac
